# Elucidating the toxicogenomic mechanisms of DBP exposure on Hepatocellular carcinoma through network toxicology and molecular docking analysis

**DOI:** 10.1371/journal.pone.0359516

**Published:** 2026-10-28

**Authors:** Dafei Dai, Nianzhao Yang, Fubao Liu, Xiaopeng Chen

**Affiliations:** 1 Department of General Surgery, The First Affiliated Hospital of Anhui Medical University, Hefei, Anhui, China; 2 Department of General Surgery, The First Affiliated Hospital of Wannan Medical College (Yijishan Hospital of Wannan Medical College), Wuhu, Anhui, China; Jamia Millia Islamia Central University: Jamia Millia Islamia, INDIA

## Abstract

**Backgroud:**

This study explored the molecular mechanisms by which dibutyl phthalate (DBP) contributes to hepatocellular carcinoma (HCC) pathogenesis using integrated network toxicology and bioinformatics.

**Methods:**

DBP’s carcinogenic potential was evaluated with ADMETLAB 3.0 and ProTox3. DBP-related targets were obtained from ChEMBL, SwissTargetPrediction, and PharmMapper; HCC-related targets from GeneCards and OMIM. Overlapping genes were identified by Venn analysis. A protein-protein interaction (PPI) network was constructed using STRING and analyzed with Cytoscape to identify hub genes. Functional enrichment analysis (GO/KEGG) was performed with DAVID. Expression validation and survival analysis used TCGA-LIHC and GEO (GSE39791) data. A prognostic nomogram integrating gene expression and clinical variables was developed. Single-cell RNA sequencing (GSE299340) was analyzed with Seurat to examine hub gene expression. Molecular docking and dynamics simulations investigated DBP interactions with core targets.

**Results:**

A total of 242 overlapping genes between DBP-associated and HCC-associated targets were identified. PPI network analysis revealed seven hub genes: KRAS, GRB2, ESR1, HSP90AA1, SRC, EGFR, and TP53. Enrichment analysis indicated significant involvement in cancer-related pathways. Expression analysis in TCGA and GEO datasets confirmed significant dysregulation of GRB2, ESR1, HSP90AA1, and SRC in HCC tissues, which correlated with patient survival outcomes. The prognostic model incorporating these genes showed good predictive accuracy. Single-cell analysis indicated distinct expression patterns of ESR1 (hepatocytes) and HSP90AA1 (immune cells) in HCC. Molecular docking demonstrated stable binding of DBP to ESR1 and HSP90AA1 with favorable binding energies (−6.8 and −6.1 kcal/mol, respectively). Molecular dynamics simulations supported the stability of these complexes, with van der Waals interactions being the primary binding force.

**Conclusions:**

This integrative analysis suggests that DBP exposure may contribute to HCC pathogenesis by modulating core genes and pathways, although these findings are associative and require experimental confirmation. Hub genes, especially ESR1 and HSP90AA1, are potential biomarkers and targets. Direct DBP-protein interactions provide mechanistic insights into HCC etiology.

## Introduction

Plastic pollution has emerged as a critical environmental concern worldwide. Current estimates indicate that global plastic production exceeds 350 million metric tons every year [1]. Phthalates are common chemical plasticizers in plastics. The addition of phthalates to plastics can improve the physical and mechanical properties of plastics. The world’s annual production of phthalates is huge. Dibutyl phthalate (DBP), a prevalent phthalate derivative in industrial formulations, demonstrates environmental ubiquity across construction materials, consumer packaging systems (including food/medical products), childcare articles, personal care formulations, and pesticidal products [2–4]. Low molecular weight phthalates like DBP can be released into the environment, including water, soil, organic sediments, air, and food, reflecting its widespread distribution in both abiotic and biotic matrices [5,6]. Given the ubiquitous presence of DBP, its safe exposure levels and potential health risks have generated significant concern.

Hepatocellular carcinoma (HCC**)** was the most prevalent type among the nearly 906,000 liver cancer cases reported worldwide in 2020 [7]. Globally, HCC ranks as the third most prevalent contributor to cancer-related mortality, demonstrating a 5-year relative survival rate of roughly 18%. The parallel trends in disease incidence and mortality, with an annual death of 830,000, emphasize the poor prognosis associated with this disease [8]. HCC progression is widely recognized as a complex process requiring prolonged carcinogenesis, which may be accelerated by exposure to genotoxic agents including both non-genotoxic promoters and DNA-damaging carcinogens [9]. Prolonged exposure to DBP could lead to imbalance of hepatic lipid homeostasis, thereby triggering toxicity and inflammatory processes [10,11]. Meanwhile, DBP can induce liver fibrosis, and its further development can contribute to the pathogenesis of cirrhosis [12]. Several experimental evidence indicates that exposure to DBP can increase the risk of progression in HCC [13,14]. Advances in exposure monitoring methods have allowed researchers to more accurately analyze the relationship between DBP exposure and HCC, enabling for an accurate risk assessment.

Conventional toxicity evaluation approaches primarily target specific toxicity endpoints, demonstrating limited capacity for comprehensive characterization of compound hazards and their multidimensional interactions within biological systems. An integrated approach combining network toxicology and molecular docking was employed to elucidate the molecular mechanism underlying DBP induced HCC pathogenesis. Network toxicology is an emerging field that uses systems-level thinking to study chemical toxicity. It draws on large datasets from genomics, proteomics, and metabolomics, alongside chemical informatics, to examine how chemicals interfere with biological pathways. The core idea is that toxicity does not arise from isolated molecular events, but rather from widespread network disruptions that impair cellular function and homeostasis, ultimately increasing the risk of disease [15]. Molecular docking emulates stereochemical binding of compounds to targets favorable binding affinities, clarifying possible molecular mechanisms by which these chemicals could promote tumorigenesis.

Through the application of these advanced methodologies, this research aims to explore the functional mechanism by which DBP may influence hepatocarcinogenesis and its clinical progression, with the goal of establishing a scientific basis for understanding its potential toxicity attributes and molecular mechanisms, while also offering academic evidence for assessing environmental contaminant toxicity and formulating preventive health strategies. We emphasize that our computational findings are hypothesis generating and intended to guide future experimental investigations.

## Methods

### Network toxicological evaluation of DBP

The chemical structure of DBP, along with its associated molecular parameters—namely, molecular formula, molecular weight, and SMILES notation—were retrieved from the PubChem database (Last update: 26 February 2025) [16]. The carcinogenic potential of DBP was further verified by employing both the ADMETLAB 3.0 system (version3.0, Last update: 31 January 2024) and the ProTox3 toxicology repository (version3.0, Last update: May 2024). The graphical abstract is drawn by Figdraw 2.0.

### Acquisition of DBP-related gene targets

Potential human gene targets of DBP were retrieved from the ChEMBL database (versionChEMBL 35, Last update: December 2024), Swiss Target Prediction database (version2019, Last update: May 2019), and PharmMapper database (version2017, Last update: 1 January 2016), using “Butyl phthalate,” “Dibutyl phthalate,” “Di-n-butyl phthalate” or “Di-n-butyl phthalate” as the search term. Following the integration of genetic datasets from the three database, redundant entries were eliminated, and standardization of target names was performed through the UniProt database (version2025_01, Last update: January 2025) [17]. Then definitive target gene set for DBP was constructed.

### Acquisition of HCC-related gene targets

The GeneCards (version5,24, Last update: March 2025) and OMIM databases (accessed on 27 March 2025) were queried with “hepatocellular carcinoma” and “hepatocellular cancer” as search terms to identify HCC-related targets. To validate the strong association between identified gene targets and HCC pathogenesis under DBP exposure, genes with a “Relevance score” ≥ 10 were selected. The targets were then merged into a unified HCC target dataset.

### The overlap analysis of DBP-related gene targets and HCC-related gene targets

The overlap analysis of DBP-related gene targets and HCC-related targets was conducted via the Venn website [18].We submitted the DBP-related genes and the HCC-related genes to an online Venn diagram tool, which automatically produced the overlap results.

### Construction of protein–protein interaction (PPI) networks

The PPI network was assembled employing the STRING database (version12.0, Released: 26 July 2023) [19] with Venn-derived intersection targets. Following the selection of Homo sapiens, a high-confidence interaction threshold of ≥0.7 was used to construct the interactome map. STRING-generated interaction datasets were integrated into Cytoscape v3.7.0 to perform topological characterization and construct visualized PPI network. Then, we utilized the CytoNCA plug-in to evaluate the topological parameters of the PPI network. Core gene targets for DBP-induced HCC were identified through a multi-centrality screening strategy, requiring nodes to exceed median thresholds for Betweenness centrality, Closeness centrality, Degree centrality, Eigenvector centrality, Local Average Connectivity (LAC), and Network centrality. Then re-import the obtained gene set to creating sub-Network. Repeat the above method 3 more times, and finally obtain the core gene targets.

### GO/KEGG Enrichment analysis

Utilizing the DAVID database (Last update: 22 December 2024), we conducted Gene Ontology (GO) annotation and Kyoto Encyclopedia of Genes and Genomes (KEGG) pathway enrichment analyses to explore the molecular mechanisms involved in HCC pathogenesis. GO term prioritization was implemented based on FDR (False Discovery Rate) thresholds, with the ten terms exhibiting minimal FDR values within each ontological category (BP/CC/MF) being displayed graphically in the enrichment results diagram. For a clearer view of the functional enrichment results, we produced visual outputs from the SRplot platform for both GO annotations and KEGG pathway analyses [18].

### Independent prognostic prediction analysis

Expression and clinical data for the seven core genes were obtained from The Cancer Genome Atlas (TCGA) database through the GDC Data Portal (version42.0, Released: 25 April 2025). To assess their combined prognostic value, a predictive nomogram was developed by integrating the gene expression profiles with key clinical variables, including patient age, gender, pathologic T-stage, tumor grade, and new tumor event status. Candidate predictors were first screened using univariate Cox regression, with only those achieving statistical significance subsequently entered into a multivariate Cox regression model to identify independent prognostic factors. The final nomogram, which incorporated variables significant in both analytical stages, was built utilizing the “rms” package in R to stratify patient risk. The results of the regression analyses, including hazard ratios (HRs) and 95% confidence intervals (CIs), were represented graphically as a forest plot using the “forestplot” package. A comprehensive comparative analysis of core gene expression levels, clinical characteristics, survival status, and follow-up time was then conducted to substantiate the utility of these genes as independent prognostic biomarkers.

### Expression and prognostic survival analyses in TCGA LIHC

Using the same TCGA-LIHC dataset described above (GDC Data Release 42.0), which offers comprehensive genetic and clinical data about these patients. Patients lacking clinical information were omitted from the analysis. All the data have been standardized. This dataset includes 369 HCC samples and 50 normal samples. Differences in core gene expression between the two groups were analyzed using the t test. The prognostic value of core genes association with HCC patient overall survival outcomes was assessed through survival analysis. Furthermore, to assess the clinical relevance of each core gene target in survival outcome prediction, the ROC curve was quantified. Violin diagram, survival analyses and ROC curves on the core genes were performed using the SRplot bioinformatics platform.

### GEO database expression validation

To identify the expression levels of core gene targets associated with HCC, the GEO database was searched, and the dataset GSE39791 (GPL10558) was selected. The GSE39791 dataset includes 72 paired hepatocellular carcinoma tissues and their adjacent tissues. Use the limma R package to process and analyze the data. Gene expression levels and ROC curves were visualized through the SRplot bioinformatics platform.

### Single-cell analysis of Core genes

To elucidate the function of hub genes in hepatocellular carcinoma (HCC) at single‑cell resolution, we performed an analysis of single‑cell RNA sequencing (scRNA‑seq) data. The study utilized the publicly available dataset GSE299340 from the GEO database [20], which includes three normal liver samples and three HCC patient samples. Preprocessing and analytical steps were carried out with the Seurat toolkit in R. Genes displaying high variability were selected for dimensionality reduction via principal component analysis (PCA), followed by cell clustering. For visualization of high‑dimensional scRNA‑seq data in two dimensions, the t‑SNE algorithm was employed. Technical batch effects between diseased and control samples were mitigated using the Harmony package, thereby integrating datasets while retaining biologically relevant variation. Cell types were annotated with the assistance of the Single R package. Finally, the expression patterns and distribution of the identified hub genes related to DBP‑HCC were evaluated across distinct cell clusters.

### Molecular docking to the core gene targets

3D structural data for core gene targets ESR1 ((PDB entry 4TV1, version 1.2, released on 2015-01-28) and HSP90AA1 (PDB entry 5J2V, version 1.3, released on 2017-10-11) were obtained from the RCSB PDB platform [21], and the chemical structure of DBP was retrieved from the PubChem database in both SMILES and 3D SDF formats. For receptor preparation, water molecules and heteroatoms not relevant to binding were removed, and polar hydrogens were added using MGLTools 1.5.7. The receptor and ligand were then converted to pdbqt format using the same tool. Docking grid boxes were defined to encompass the entire protein, with the following parameters: for ESR1 – center_x = 11.305, center_y = 8.800, center z = 21.615, size x = 62.584, size y = 50.231, size z = 67.454, exhaustiveness = 8; for HSP90AA1 – center x = –33.930, center y = 14.313, center z = –21.107, size x = 47.922, size y = 45.866, size z = 46.531, exhaustiveness = 8. Molecular docking was performed using AutoDock Vina 1.2.5. The resulting poses were clustered by RMSD (threshold 2.0 Å), and the cluster with the lowest binding energy was retained for each target. Binding affinities ≤ –5.0 kcal/mol were considered indicative of significant molecular recognition. No co‑crystallized reference ligand was available for these PDB entries; therefore, classical redocking validation could not be performed. Instead, the reliability of the docking predictions was further assessed by 100‑ns molecular dynamics simulations. Finally, Discovery Studio was used for visualization and analysis of the docking poses.

### Molecular dynamics simulation

To explore the stability of the protein–ligand interactions in greater depth, molecular dynamics simulations were performed on two complexes: DBP-ESR1 and DBP-HSP90AA1 using GROMACS 2022 software. The GAFF force field was selected for the small molecule ligands, and the AMBER14SB force field coupled with the TIP3P water model was employed for the protein. The complete simulation system was then established through the integration of all components. It is carried out under constant temperature, constant pressure and periodic boundary conditions. The LINCS algorithm constrained all hydrogen bonds using a 2 fs timestep. For electrostatic calculations, the Particle-mesh Ewald (PME) method was employed with a 1.2 nm cutoff. Van der Waals interactions were truncated at 10 Å and updated every 10 steps. Temperature and pressure were maintained at 298 K and 1 bar using the V-rescale and Berendsen coupling methods, respectively. The composite system was subjected to a 100 ps NVT simulation, a 100 ps NPT simulation, and finally a 100 ns MD simulation at 298 K; conformational snapshots were saved every 10 ps throughout the production run. Following the completion of the simulation, the trajectories were processed using VMD and Pymol, while the MM‑GBSA binding free energy for the protein‑ligand complex was evaluated using the g_mmpbsa program.

## Results

### Toxicity assessment of DBP

DBP’s toxicity was analyzed using two computational toxicology platforms, applying a consensus approach where positive carcinogenicity prediction from either tool led to classification as toxic. Ultimately, DBP passed the toxicity test in both databases (Table 1).

**Table 1:** Molecular parameters and carcinogenicity of DBP.

Name	Molecular formula	Molecular weight(g/mol)	SMILES structures	Carcinogenicity	
				admetlab3.0	ProTox3
DBP	C16H22O4	278.34	CCCCOC(=O)C1 = CC = CC = C1C(=O)OCCCC	0.714	0.58

### Identification of gene targets of DBP-related HCC

Using stringent filters, we retrieved 2034 HCC-related gene targets from GeneCards and 315 from OMIM. After filtering out overlapping genes, 2258 unique HCC-related gene targets were obtained (S1 Table) (Fig 1. A). A total of 266, 101 and 287 DBP-related gene targets were retrieved from ChEMBL, Swiss Target Prediction, and PharmMapper database respectively. Through deduplication procedures, 611 unique DBP-related gene targets were obtained (S2 Table) (Fig 1B). By meticulously datasets integration and redundancy removal, 242 overlapping gene targets were pinpointed (S3 Table) (Fig 1C, D).

**Fig 1:**
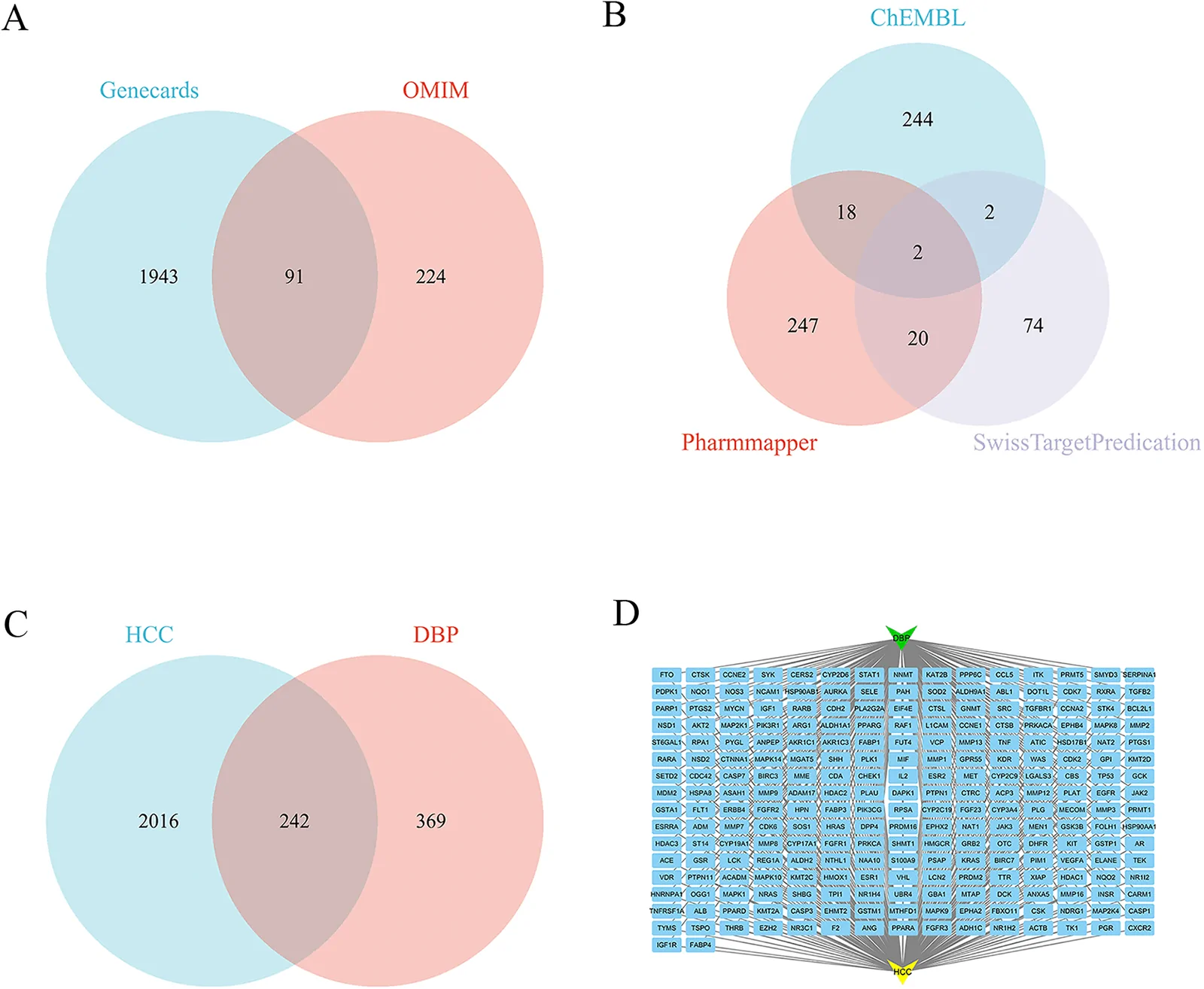
Venn diagram of the targets of HCC (A), DBP (B), HCC-DBP (C) and DBP-target-HCC network diagram (D).

### Potential targets with the PPI network

Bioinformatic analysis via STRING generated a PPI network containing 242 nodes and 1317 edges, with a mean nodal connectivity of 10.9 (Fig 2A). Subsequently, the results were subjected to topological analysis in Cytoscape 3.7.0. Based on comprehensive topological metrics comprising Betweenness centrality, Closeness centrality, Degree centrality, Eigenvector centrality, LAC, Network centrality, we identified seven core gene targets: KRAS, GRB2, ESR1, HSP90AA1, SRC, EGFR, and TP53 (Table 2) (Fig 2B–E).

**Table 2:** The seven targets screened in the PPI network.

Gene	Betweenness	Closeness	Degree	Eigenvector	LAC	Network
KRAS	7.971	0.786	16	0.234	11.250	13.827
GRB2	4.959	0.759	15	0.225	11.200	13.018
ESR1	13.148	0.815	17	0.237	10.588	14.024
HSP90AA1	16.469	0.880	19	0.264	12.000	17.397
SRC	24.108	0.957	21	0.284	12.476	20.087
EGFR	26.962	0.957	21	0.279	11.905	19.666
TP53	23.744	0.957	21	0.283	12.476	20.232

**Fig 2:**
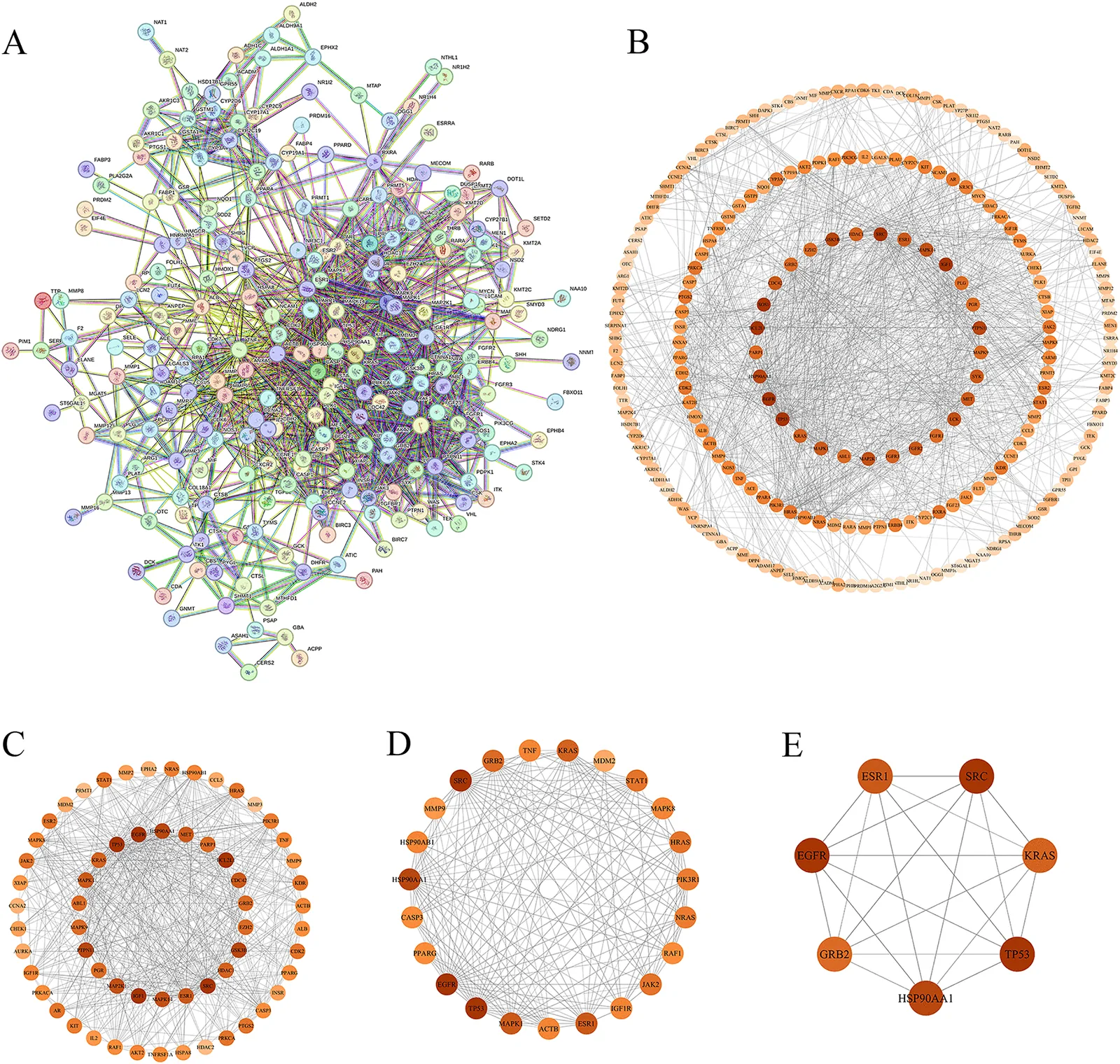
Identification of core gene targets and functional interaction network diagram. (A) PPI network analysis of DEGs (STRING database); (B, C, D, E) The top 7 Hub genes identified by six centrality algorithms via the CytoNCA plug-in in Cytoscape, Fig 2.C, Fig 2. D and Fig 2. E is sub-networks respectively. Nodes are colored according to their degree values, with darker colors and larger circles indicating stronger interactions.

### GO/KEGG enrichment analysis

Bioinformatic analysis through GO term enrichment established that these differentially expressed genes are predominant involvement in biological processes (BP) such as negative regulation of apoptotic process and insulin-like growth factor receptor signaling pathway and chromatin remodeling, which are crucial for modulating cell growth and cell proliferation. The cellular component (CC) enrichment highlights elements including the cytosol, extracellular exosome, cytoplasm and nucleus, which are crucial to cellular trafficking and signal transduction. The molecular function (MF) analysis reveals an enrichment of genes associated with molecular binding, such as enzyme binding, histone H3Y41 kinase activity, histone H2AXY142 kinase activity, identical protein binding, which is recognized as a critical regulator in signaling pathways that modulate cell proliferation and viability (Fig 3A). Meanwhile, KEGG pathway analysis identified marked enrichment in critical signaling pathways, with notable emphasis on cancer-related pathways, such as pathways in cancer, prostate cancer, proteoglycans in cancer, lipid and atherosclerosis (Fig 3B). These findings imply that DBP could potentially mediate cancer pathogenesis by orchestrating molecular alterations in tumorigenesis-associated signaling cascades.

**Fig 3:**
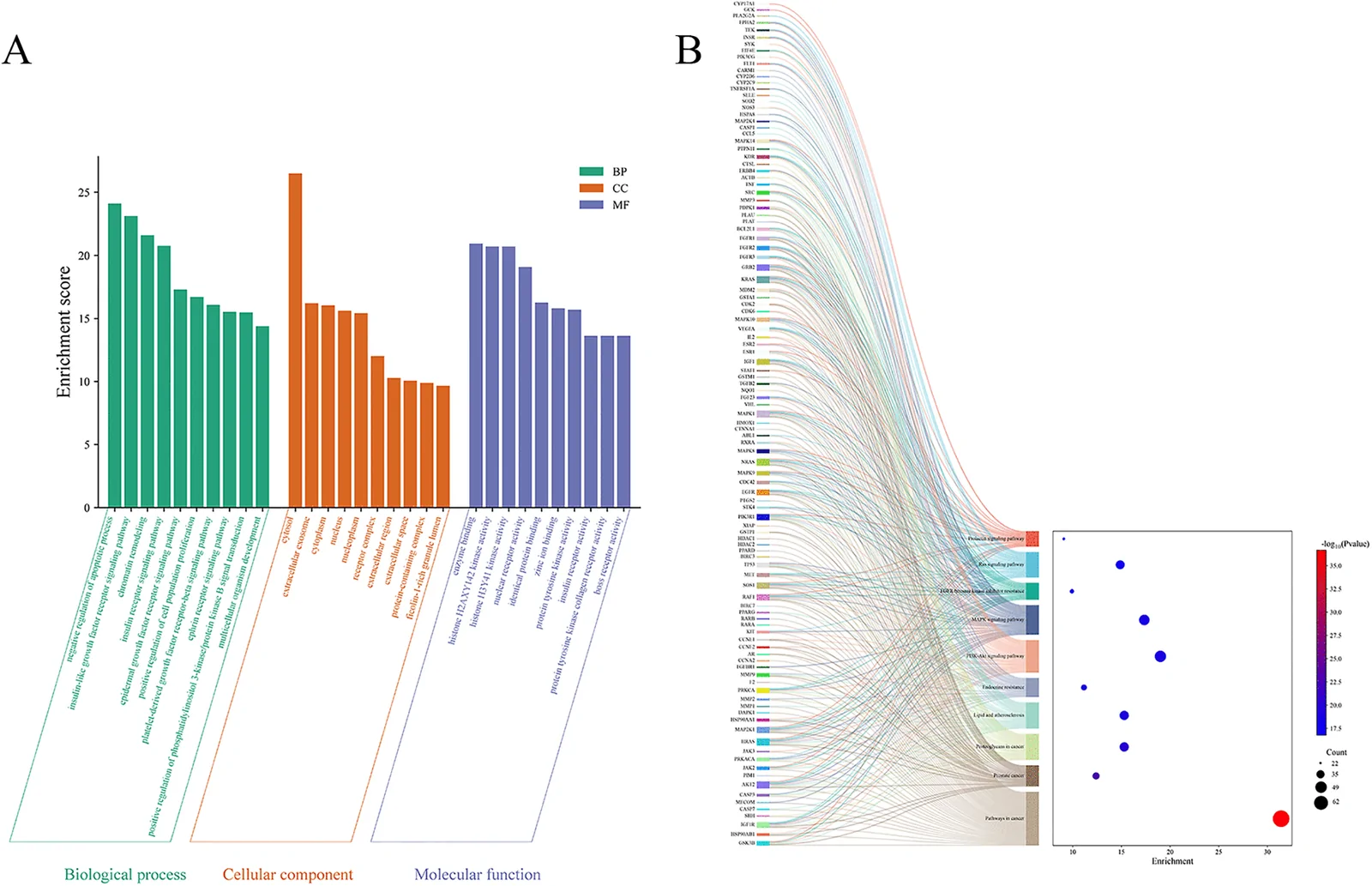
GO/KEGG results (A) GO analysis of potential targets. (B) Sankey Diagram Visualizing the Interconnectivity of Key Pathways and Genes Underlying the effect of DBP on HCC.

### Construction and validation of prognostic models

To determine its independent predictive value, the model was assessed alongside standard clinical variables from the TCGA LIHC dataset. Univariate and subsequent multivariate Cox regression analyses were conducted on the five core genes, age, gender, pT-stage, tumor grade, and new tumor event status (Fig 4A, B). The analyses identified HSP90AA1, ESR1, and pT-stage as significant independent prognostic factors, with each achieving a P-value of less than 0.05. This confirms that these two genes and pT-stage provide prognostic information independent of other clinical features in hepatocellular carcinoma.

**Fig 4:**
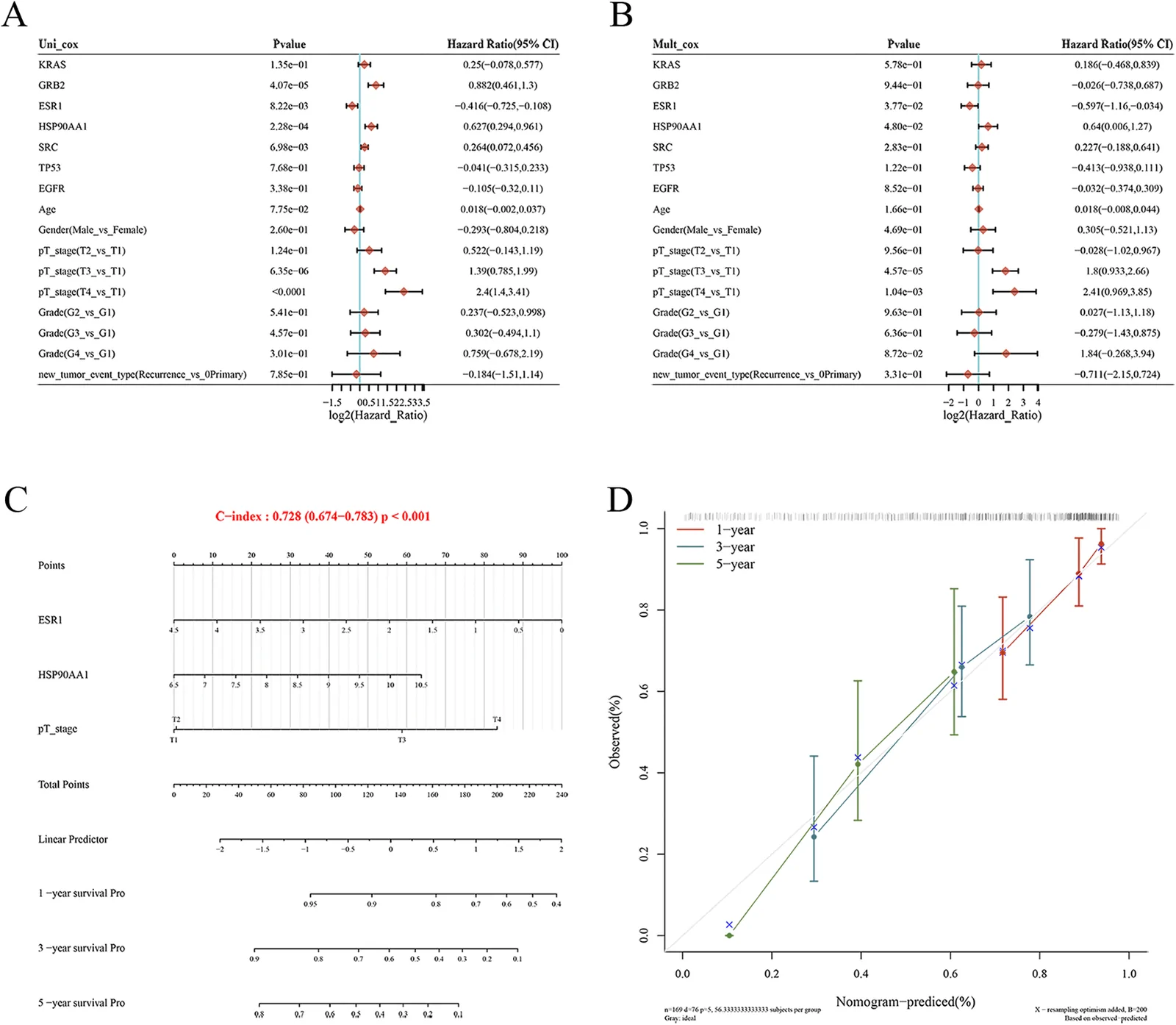
Independent prognostic predicting ability of the model. (A) Multivariate Cox regression was used to evaluate the risk coefficient and confidence interval; (B) Univariate Cox regression analysis assessed the risk coefficient and confidence interval; (C) A nomogram was developed based on two core genes and five clinicopathological features in the TCGA-LIHC cohort; (D) Calibration curves for risk prediction of the overall survival nomogram model are presented for 1-year, 3-year, and 5-year time points.

To develop a clinically applicable prognostic model, a nomogram was constructed using the TCGA-LIHC cohort, integrating a molecular risk score derived from core genes with key clinicopathological parameters. This tool enables the prediction of individual patient survival probabilities. Analysis revealed that the contribution of individual clinical factors and the expression levels of the five core genes to the overall risk score varied across the samples. The model’s predictive accuracy for 1-, 3-, and 5-year survival was evaluated using calibration curves, which demonstrated a middle degree of concordance between predicted and observed outcomes (Fig 4C, D). These results suggest the model’s potential for standalone prognostic application.

### Expression and prognostic survival analyses

To validate the expression profiles and prognostic significance of the core gene targets, we utilized the GSE39791 and TCGA LIHC datasets. Our analysis revealed that both ESR1 and HSP90AA1 exhibited statistically significant expression levels (P < 0.001), as illustrated in Fig 5A, C. Notably, ESR1 was downregulated in tumor tissues, whereas HSP90AA1 showed marked upregulation. The diagnostic potential of these genes was further supported by ROC curve analysis: in the GSE39791 dataset, the area under the curve (AUC) values for ESR1 and HSP90AA1 were 0.893 and 0.878, respectively (Fig 5B), while in TCGA LIHC cohort, they were 0.915 and 0.889, respectively (Fig 5D). Survival analysis demonstrated that elevated ESR1 expression was robustly associated with prolonged overall survival (P = 0.00013, Fig 5E). Conversely, high HSP90AA1 expression correlated with significantly poorer survival outcomes (P = 0.00018, Fig 5F).

**Fig 5:**
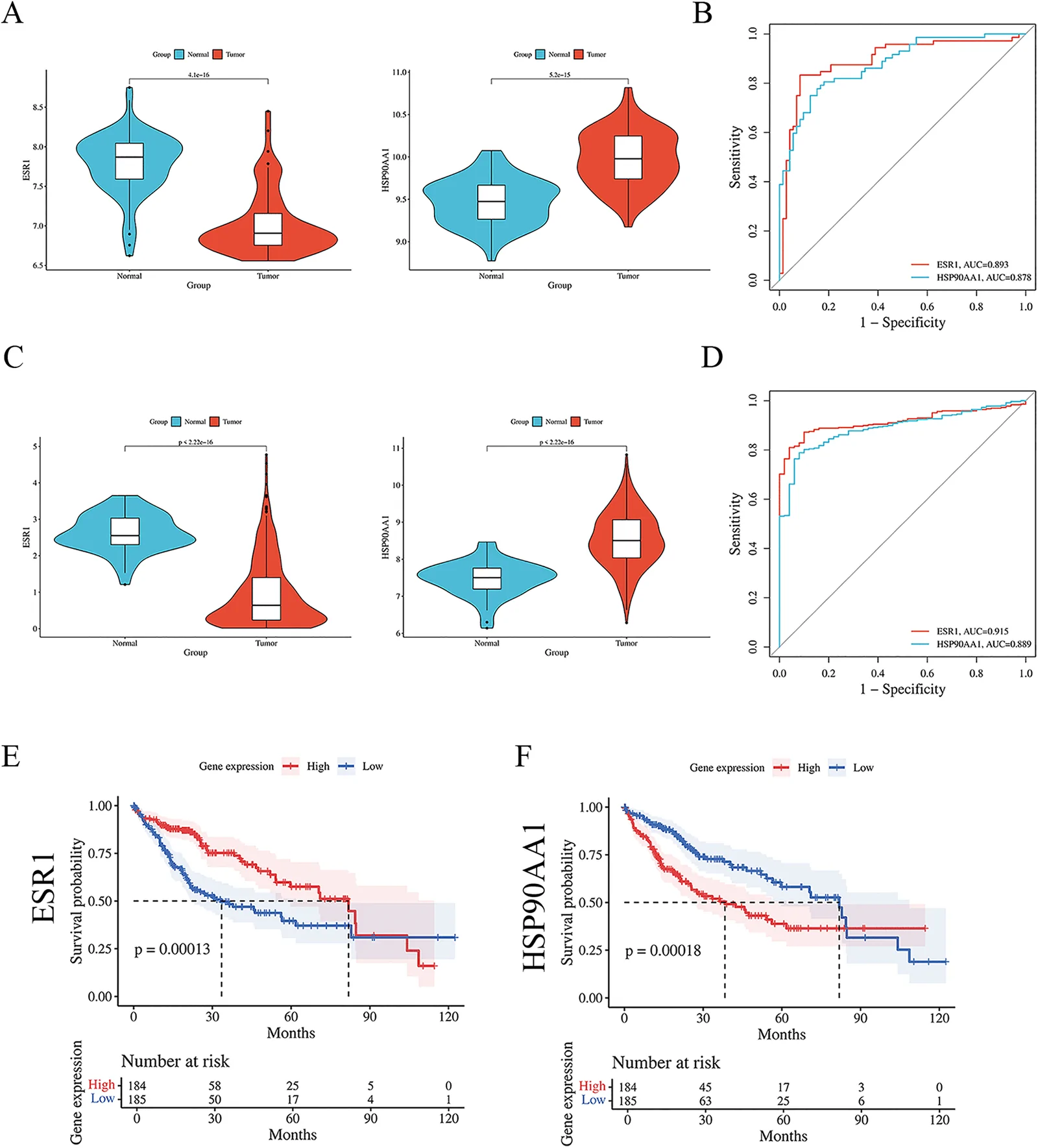
Analytical evaluation of core targets in GSE39791 and TCGA LIHC. (A) Expression analysis of core genes in the GSE39791 dataset; (B) ROC curves of core genes in the GSE39791 dataset; (C) Expression analysis of core genes in the TCGA-LIHC dataset; (D) ROC curves of core genes in the TCGA-LIHC dataset; (E) Prognostic survival analysis of ESR1 in the TCGA-LIHC dataset, (F) Prognostic survival analysis of HSP90AA1 in the TCGA-LIHC dataset.

### Expression of genes in single cells

The GSE299340 single-cell RNA sequencing dataset was processed using the Seurat pipeline in R. Following quality control steps, we assessed cellular heterogeneity by performing principal component analysis (PCA) and t-distributed stochastic neighbor embedding (t-SNE), utilizing 1,500 genes characterized by pronounced transcriptional variability (Fig 6A, B). To determine cell identities, we applied the SingleR reference-based annotation tool, which categorized the cells into eleven distinct lineages: Hepatocytes, Endothelial cells, Fibroblasts, B cells, CD8 + T cells, T cells, NK cells, Monocytes, Macrophages, Dendritic cells, and Neutrophils (Fig 6C). When comparing HCC specimens with normal liver tissues, we observed substantial differences in the relative frequencies of multiple cell subsets.

**Fig 6:**
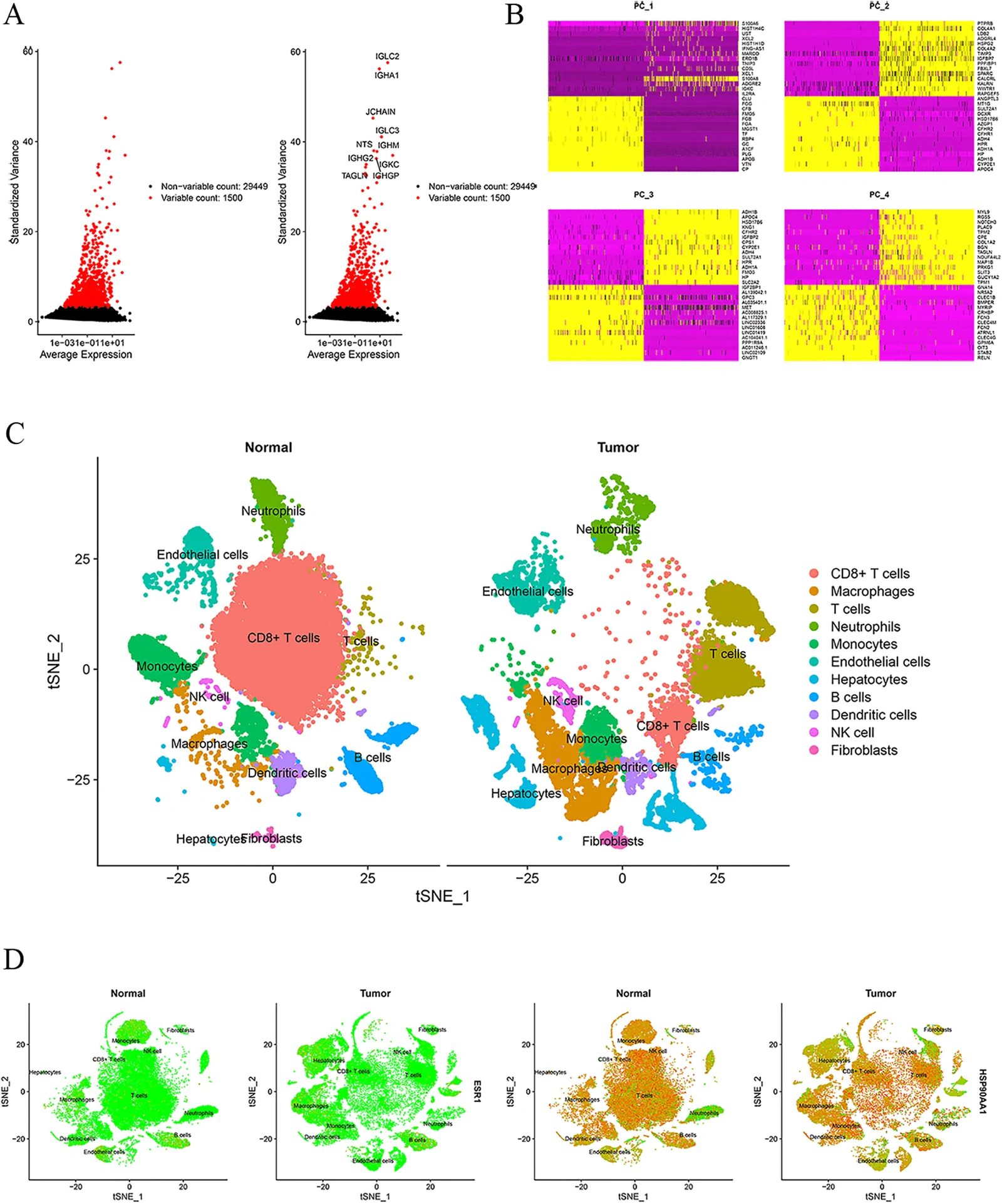
Examination of the single-cell expression profiles of hub genes in HCC. (A) Detection of genes exhibiting pronounced expression variability in HCC scRNA-seq dataset; (B) Visualization of the top four principal components via heatmap based on HCC scRNA-seq data; (C) Clustree analysis illustrating clustering results for normal and tumor samples; (D) Display of hub DBP-HCC expression patterns within the HCC scRNA-seq data.

Subsequently, we examined the distribution of hub gene expression across these defined cellular compartments. The data indicated that ESR1 transcripts were confined principally within Hepatocytes, where they attained peak levels (Fig 6D). Conversely, HSP90AA1 exhibited a widespread presence across multiple lineages; however, its expression was substantially upregulated in CD8 + T cells and T cells within the tumoral microenvironment (Fig 6D), highlighting a pronounced disparity relative to normal liver tissue.

### Molecular docking

The molecular docking findings revealed that DBP could form stable interactions to the binding sites of ESR1 and HSP90AA1, exhibiting low binding energies (− 6.8 and − 6.1 kcal/mol, respectively), demonstrating a significant molecular recognition. Notably, the DBP-binding site in ESR1 is located within the canonical ligand-binding domain, a functionally relevant region where endogenous estrogens and known modulators interact. For HSP90AA1, DBP occupied the N-terminal ATP-binding pocket, which is a well-characterized regulatory hotspot targeted by various small-molecule inhibitors. Key binding sites for ESR1 and DBP included LEU, ALA, PHE and MET (Fig 7A). For HSP90AA1, DBP binding sites were THR, ASN, VAL and LEU (Fig 7B),

**Fig 7:**
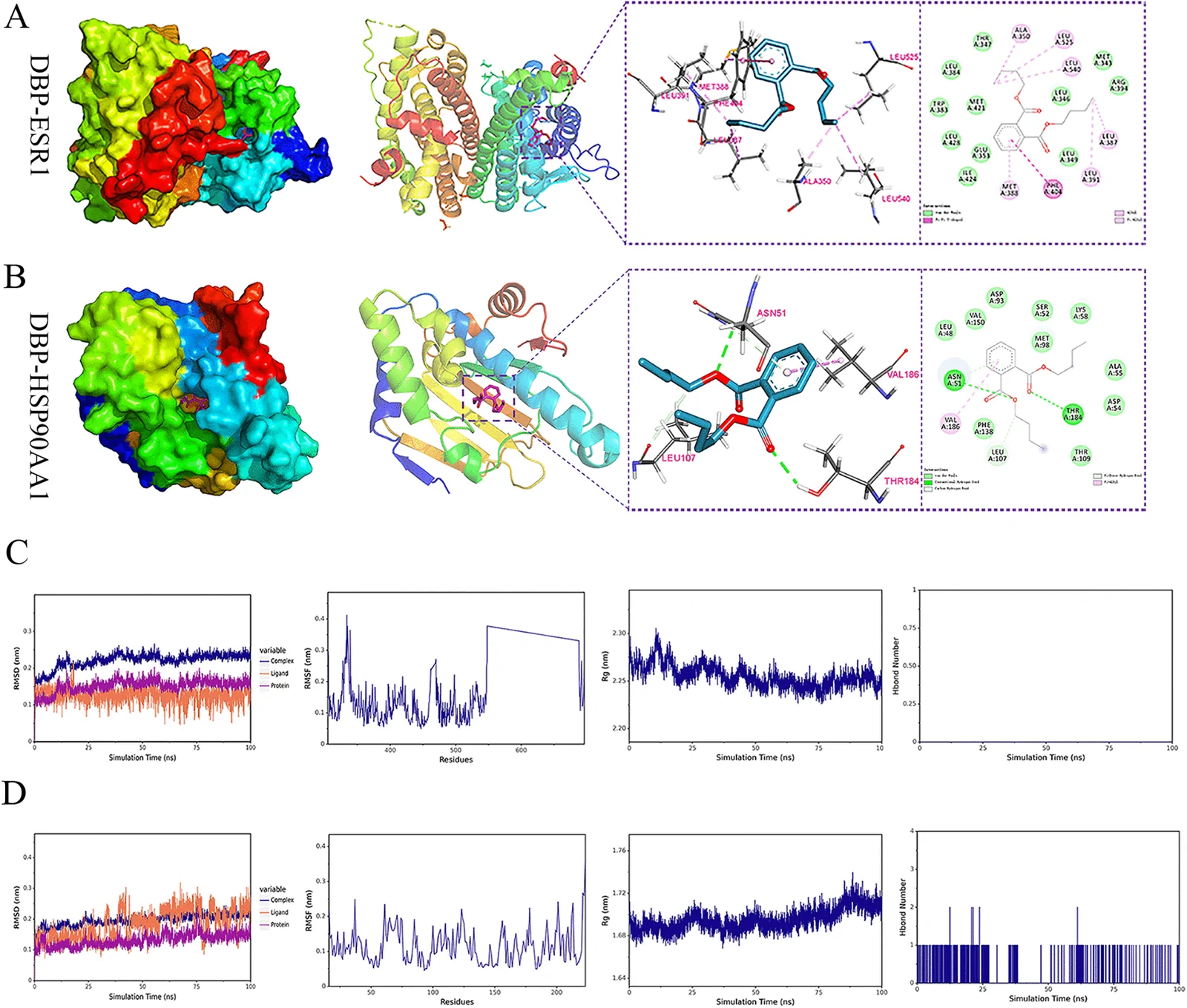
Molecular docking and MDS results of DBP with core target proteins, displaying the lowest binding energies in both 3D and 2D formats. (A) Docking of DBP with ESR1; (B) Docking of DBP with HSP90AA1; (C) MD simulation analysis of DBP with ESR1; (D) MD simulation analysis of DBP with HSP90AA1.

The results identify essential amino acid residues involved in the interaction of proteins with DBP. This demonstrates that DBP could engage in direct molecular interactions with core gene targets, thereby modulating HCC-associated molecular mechanisms. Subsequent investigation established that DBP formed a minimum of two hydrogen-bonding interactions with key residues in HSP90AA1, highlighting robust intermolecular interactions. These results reveal that the tight interaction of DBP to these core receptor targets could be pivotal in the molecular mechanisms of HCC during prolonged exposure.

### Molecular dynamics simulations

The conformational dynamics and stability of the DBP-core gene target complexes were elucidated through a 100-ns all-atom molecular dynamics simulation performed in GROMACS. Post‑simulation, the resultant trajectories were analyzed with the program’s native utilities to determine the RMSD, RMSF, Rg, hydrogen bond number, and the MM‑GBSA binding free energies. To assess the reliability of these estimates, we performed block‑averaging analysis of the RMSD trajectories over successive 10‑ns windows. This analysis indicated that the structural fluctuations reached a relatively stable plateau during the latter half of the simulation, supporting the convergence of our measurements. Standard errors of the mean for RMSD, RMSF, and Rg were calculated from the trajectory frames and are reported alongside the mean values.

The RMSD map of the complex formed by DBP and ESR1 and HSP90AA1 fluctuate relatively little, implying stable RMSD values for these core gene targets (Fig 7C, D). The RMSF value provides an in-depth understanding of the flexible movement and structural changes of specific residues. The Fig shows that the RMSF value varies between 0.05 and 0.2 nm (Fig 7C, D). The Radius of Gyration (Rg) describes the spatial distribution of a molecule’s atoms around its center of mass, serving as a key metric for quantifying the overall compactness of a protein-small molecule complex. The structural integrity of the complexes, as evidenced by a steady Rg throughout the simulation, was consistent with the RMSD data observed (Fig 7C, D). Except for ESR1 which did not form hydrogen bonds with DBP, the hydrogen bond count for HSP90AA1-DBP complexes primarily varied between 1 and 2 (Fig 7C, D). To assess the consistency between the docking and molecular dynamics results, we compared the binding modes obtained from the initial docking poses with those sampled during the molecular dynamics trajectories. For both complexes, the key contacting residues identified in the docking poses remained involved in stabilizing the interactions throughout the simulations, with the overall binding geometry largely preserved. The MM‑GBSA binding energies (Table 3) are consistent with the docking scores, and van der Waals interactions dominate the binding in both cases. Regarding the energy components, as shown in Table 3, the van der Waals contribution (ΔEvdw) was larger than the non‑polar solvation term (ΔGnonpol) in all complexes and both were significantly greater than the electrostatic component (ΔEele). Consequently, van der Waals forces are the primary contributor to the total binding energy, followed by non-polar interactions, while electrostatic forces play a supplementary role. The binding energy and affinity of the DBP-protein complexes are both relatively high. Overall, the interaction complexes formed between DBP and all core gene targets demonstrated favorable kinetic properties, which again corroborated the reliability of the molecular docking results.

**Table 3:** The binding free energy components (ΔGMM-GBSA) of DBP with core targets calculated by MM-GBSA method (kJ/mol).

Complex	ΔEvdw	ΔEele	ΔGpol	ΔGnonpol	ΔGMM-GBSA
ESR1-DBP	−187.388 ± 0.195	−4.163 ± 0.336	43.373 ± 0.758	−21.007 ± 0.086	−169.184 ± 0.794
HSP90AA1-DBP	−146.138 ± 2.186	1.428 ± 0.903	54.891 ± 2.143	−21.446 ± 0.192	−111.264 ± 2.572
ΔGMM-GBSA = ΔEele + ΔEvdw + ΔGpol+ΔGnonpol					

## Discussions

The present research implemented a network toxicology methodology, synthesizing data from ChEMBL, Swiss Target Prediction, PharmMapper, GeneCards and OMIM databases to exhaustively delineate 242 candidate targets potentially linked to DBP exposure and HCC pathogenesis. Employing the STRING platform, Cytoscape software, GEO data and TCGA LIHC data, we established a PPI network and seven core gene targets (KRAS, GRB2, ESR1, HSP90AA1, SRC, EGFR, and TP53) were selected.

Our integrative framework spans multiple scales: from molecular docking (predicting direct DBP‑protein interactions), through tissue‑level transcriptomics (TCGA and GEO cohorts), to single‑cell resolution (scRNA‑seq). This multi‑layered design allows us not only to identify candidate targets but also to infer the cellular contexts in which DBP might exert its effects—contexts that may differ substantially between hepatocytes and immune cells.

Prognostic models, expression analysis, prognostic survival analysis, and ROC curve analysis further confirmed that these two core gene targets—ESR1 and HSP90AA1—not only demonstrate pronounced associations with HCC progression but also serve as robust biomarkers for survival prediction. This differential gene regulation pattern suggests a possible DBP-mediated metabolic reprogramming in HCC that may occur through selective pathway manipulation, potentially disrupting lipid metabolism, growth signaling, and stress response mechanisms.

Estrogen signaling is mediated through ERα (ESR1-encoded) and ERβ (ESR2-derived) receptors, which exhibit distinct ligand-binding specificities [22]. ESR1 is the dominant subtype of ER in liver tissue, which is the main organ of estrogen metabolism [23,24]. ESR1 is critical for regulating cell proliferation, migration, invasion [25] and has been reported to act as a tumor suppressor gene in HCC [26]. Studies have reported that ESR1 directly modulates the obesogenic metabolism-associated gene MMAA to attenuate HCC progression [27]. Upregulation of ESR1 triggered apoptosis in Hep3B through ligand-independent transcriptional activation of TNFα [28]. Moreover, Overexpression of ESR1 can regulate the expression of HS1 BP3 to inhibit HCC proliferation [29]. Large-scale TMA analysis of 339 HCC patient tumor tissues identified ERα expression in 32 cases (9.4%) and exhibited a positive relationship with increased overall survival and prolonged disease-free survival [30], which was consistent with bioinformatics results [31]. Importantly, our docking and simulation data show that DBP can directly engage the ESR1 ligand-binding pocket with appreciable affinity, raising the possibility that this environmental plasticizer may interfere with estrogen-dependent tumor-suppressive signaling, either by competing with endogenous ligands or by inducing conformational changes that alter transcriptional activity.

HSP90AA1 serves as a highly conserved molecular chaperone protein in eukaryotic species evolution [32], contains three primary regions: the N-terminal region, middle region, and C-terminal region [33]. It is essential for cellular processes including tumor cell proliferation, differentiation, migration, angiogenesis and survival [33]. Knockdown of HSP90AA1 decreased cell viability and clonal formation in HCC cells, and elevated HSP90AA1 expression in HCC was associated with a markedly inferior prognosis relative to cases with lower expression levels, consistently indicating its role in promoting tumorigenesis [34]. Studies demonstrate that elevated expression of HSP90AA1 promotes metastatic progression in ERS hepatoma cells and diminishes the apoptotic efficacy of Lenvatinib [35]. HSP90AA1 demonstrates increased upregulation in patients at advanced stages of HCC versus early stages [36], indicating a worse prognosis, which consistent with another multicenter result [37]. Our computational analyses further demonstrate that DBP forms stable interactions with HSP90AA1, with binding energies and hydrogen‑bonding patterns suggestive of a functionally relevant association. Although these in silico findings do not prove causation, they provide a testable hypothesis: chronic DBP exposure might stabilize or dysregulate HSP90AA1 client‑protein networks, thereby amplifying pro‑oncogenic signaling cascades in susceptible hepatic microenvironments.

When we examined the cellular distribution of these two targets using scRNA‑seq, a functionally informative pattern emerged. ESR1 transcripts were confined almost exclusively to hepatocytes—the primary parenchymal cells in which HCC originates. This hepatocyte‑restricted expression is consistent with a model in which DBP directly engages ESR1 within liver epithelial cells, potentially interfering with its documented tumor‑suppressive functions (e.g., transcriptional control of metabolic and apoptotic genes). In contrast, HSP90AA1 showed broad expression across multiple lineages, but its most pronounced upregulation in HCC occurred within CD8 ⁺ T cells and other T‑cell subsets, rather than in malignant cells themselves. This observation shifts the mechanistic focus: rather than acting solely as a cell‑autonomous oncogenic driver in hepatocytes, HSP90AA1 may also contribute to HCC progression by modulating stress responses and protein homeostasis in tumor‑infiltrating lymphocytes, thereby shaping the immune microenvironment.

Taken together, these compartment‑specific expression patterns suggest that DBP’s hepatocarcinogenic influence may operate through at least two convergent routes: a direct, hepatocyte‑intrinsic pathway involving ESR1‑mediated tumor suppression, and an indirect, immune‑mediated pathway involving HSP90AA1‑dependent chaperone functions in T cells. These routes are not mutually exclusive; indeed, chronic xenobiotic exposure could simultaneously perturb both compartments, creating a permissive environment for HCC initiation and progression. Our scRNA‑seq data, therefore, do not merely confirm the relevance of ESR1 and HSP90AA1—they refine the cellular context in which future mechanistic and therapeutic studies should be directed.

To further explore the potential biological impact of DBP exposure, molecular docking and molecular dynamics simulations were conducted to evaluate its possible interactions with ESR1 and HSP90AA1. Both targets exhibited binding energies below −5 kcal/mol, and subsequent dynamics simulations suggested the stability of these interactions. This thermodynamic evidence is consistent with the idea that DBP could form stable complexes with these HCC-associated proteins.

However, several methodological limitations warrant caution in interpreting these computational findings. First, target prediction is inherently algorithm-dependent and may inherit biases from incomplete or noisy databases, increasing the risk of false-positive or false-negative associations. For instance, the DBP-HCC interactions inferred through network analysis require rigorous experimental validation to establish biological relevance. Second, computational models cannot fully capture the complexity of in vivo systems—including metabolic dynamics, genetic heterogeneity, and tissue-specific microenvironments—leaving mechanistic inferences about DBP’s role in HCC provisional without laboratory confirmation. Third, our molecular dynamics simulations were carried out using a single 100‑ns trajectory per complex, without replicate runs or longer sampling. We did perform convergence checks and calculated uncertainty measures where possible, but we recognize that these do not replace the statistical strength of independent replicates. This remains a limitation of the current work. In addition, our characterization of DBP-protein interactions is currently based on overall binding energies and the main contacting residues. A more detailed residue‑level analysis and energy decomposition would certainly add mechanistic depth. However, software availability and computational capacity prevented us from conducting these analyses at this stage. Meanwhile, our docking analysis was not cross‑validated with an independent software tool, nor was it verified by re‑docking against co‑crystallized ligands. Fourth, current toxicity assessments are predominantly confined to cellular models and do not account for systemic molecular perturbations; moreover, animal studies have yet to define a clear dose-response relationship between chronic, environmentally relevant DBP exposure and HCC outcomes, raising concerns about extrapolating findings from high-dose, short-term experiments to human health risks. Finally, unmeasured confounders—such as lifestyle factors and coexposure to other environmental pollutants—may distort estimates of DBP’s carcinogenic potential, even when network toxicology adjusts for variables like age or genetic background.

Addressing these gaps will require targeted experimental studies that both validate predicted DBP-target interactions and clarify their functional consequences within HCC-related pathways. Only through such integrated, multi-evidence approaches can we more reliably assess the carcinogenic risks posed by chronic, low-level DBP exposure and move toward a more complete understanding of its mechanistic role in liver cancer.

## Conclusion

By merging network toxicology, molecular docking, and machine learning approaches, the study sought to explore the potential connection between DBP and HCC. A total of 242 potential DBP-related HCC gene targets were identified, with two core gene targets (ESR1 and HSP90AA1) emerging as candidate mediators in possible DBP-induced HCC pathogenesis. Molecular docking and Molecular dynamics simulations suggested stable binding interactions between DBP and the core proteins, providing preliminary mechanistic insights into their potential functional roles. Furthermore, our findings highlight the critical engagement of oncogenic signaling pathways, particularly those implicated in cancer pathophysiology, suggesting that DBP might disrupt metabolic reprogramming in HCC. This work refines toxicity assessment frameworks for environmental contaminants while establishing a roadmap for future studies to experimentally test therapeutic interventions aimed at counteracting DBP-associated pathological cascades.

## Supporting information

S1 Fig Graphical abstract. (TIF)

S1 Table HCC‑related gene targets retrieved from GeneCards and OMIM databases. (XLSX)

S2 Table DBP‑related gene targets retrieved from ChEMBL, Swiss Target Prediction, and PharmMapper databases. (XLSX)

S3 Table Overlapping gene targets between DBP- related and HCC- related datasets. (XLSX)
